# Gut Microbiota Composition in Rats Consuming Sucralose or Rebaudioside A at Recommended Doses Under Two Dietary Interventions

**DOI:** 10.3390/metabo15080529

**Published:** 2025-08-04

**Authors:** Meztli Ramos-García, Alma Delia Genis-Mendoza, Carlos García-Vázquez, José Jaime Martínez-Magaña, Viridiana Olvera-Hernández, Mirian Carolina Martínez-López, Juan Cuauhtémoc Díaz-Zagoya, Carina Shianya Alvarez-Villagomez, Isela Esther Juárez-Rojop, Humberto Nicolini, Jorge Luis Ble-Castillo

**Affiliations:** 1 División Académica de Ciencias de la Salud (DACS), Universidad Juárez Autónoma de Tabasco (UJAT), Villahermosa 86150, Mexico; meztli.ramos@ujat.mx (M.R.-G.);; 2 Instituto Nacional de Medicina Genómica (INMEGEN), Ciudad de México 14610, Mexico; 3 Hospital Psiquiátrico Infantil Dr. Juan N. Navarro (HPIJNN), Secretaría de Salud, Ciudad de México 14080, Mexico; 4 División de Investigación, Facultad de Medicina, Universidad Nacional Autónoma de México (UNAM), Ciudad de México 04360, Mexico; 5 División Académica de Ciencias Biológicas (DACBIOL), UJAT, Villahermosa 86150, Mexico

**Keywords:** non-nutritive sweeteners, sucralose, rebaudioside A, high-fat diet, gut microbiota

## Abstract

**Background**: Artificial non-nutritive sweeteners (NNSs), such as sucralose, have been associated with gut microbiota (GM) alterations. However, the impact of rebaudioside A (reb A), a natural NNS, on GM has received limited scrutiny. Objective: The objective of this study was to examine the response of GM composition to sucralose and reb A in rats under two dietary conditions. **Methods**: Male Wistar rats (150–200 g) fed with a normal diet (ND) or a high-fat diet (HFD) were randomly assigned to receive sucralose (SCL), reb A (REB), glucose (GLU, control), or sucrose (SUC). The NNS interventions were administered in water at doses equivalent to the acceptable daily intake (ADI). After eight weeks, the GM composition in fecal samples was analyzed through 16S ribosomal RNA gene sequencing. **Results**: The NNSs did not modify the diversity, structure, phylum-level composition, or Firmicutes/Bacteroidetes (F/B) ratio of the GM in rats under ND or HFD. However, REB with HFD decreased *Bacilli* and increased *Faecalibacterium* abundance at the class level. SCL and REB in rats receiving ND reduced the genera *Romboutsia* and *Lactobacillus*. **Conclusions**: Our study suggests that when sucralose or reb A is consumed at recommended doses, there is no alteration in the diversity or the composition of the GM at the phylum level. The clinical relevance of these findings lies in the potential modifications of the GM at specific taxonomic levels by the consumption of these NNSs. Further research involving humans and including a broader range of microbial analyses is warranted.

## 1. Introduction

Non-nutritive sweeteners (NNSs) are food additives that provide high potency in sweetness but have low or null caloric content [1]. Sucralose and rebaudioside A (reb A) are two widely consumed NNSs [2,3]. Sucralose is a synthetic disaccharide derived from sucrose and exists in over 4500 products, which represent 62% of the global market for artificial sweeteners [4]. Meanwhile, reb A, of natural origin, is the most abundant steviol glycoside that has been introduced recently into the market [5]. The Food and Drug Administration (FDA) has established the acceptable daily intake (ADI) of sucralose as 5 mg/kg body weight (BW) and as 4 mg/kg BW/day expressed as steviol equivalents for reb A [6].

In a previous study by our group, various NNSs were investigated to evaluate their effects on fasting biochemical markers and glycemic response in two groups of animals receiving different diets. For this complementary study, two NNS groups, sucralose and reb A, were selected to analyze their effects on gut microbiota composition (GM) in contrast with glucose and sucrose groups used as comparators. We aimed to determine whether the microbial changes would be associated with the metabolic outcomes previously observed. These two NNSs were chosen due to their differences in chemical structure, absorption profiles, metabolism, and microbial interactions.

Sucralose is largely not metabolized, while reb A is partially metabolized by ileal or colonic bacteria that express β-glucosidases, such as *Bacteroides* [1,7]. Thus, sucralose behaves more like a xenobiotic, resisting digestion and potentially disrupting the microbial ecosystem. Some studies in mice have reported reductions in beneficial taxa such as *Lactobacillus* and *Bifidobacterium*, accompanied by increases in potentially harmful bacteria such as *Proteobacteria*, a phylum associated with inflammatory states [4]. Furthermore, others have observed glucose intolerance induction mediated by altered intestinal microbiota after 11 weeks of sucralose treatment in mice [8]. Even a low dose of sucralose (0.0003 mg/mL) administered over 16 weeks increased *Tenacibaculum*, *Ruegeria*, *Staphylococcus*, and *Allobaculum* proportions in the mouse jejunum, ileum, and colon [9]. Potential mechanisms by which GM is modulated by sucralose in animal models include an increase in intestinal permeability (leaky gut) [10,11]; meanwhile, reb A has not shown this effect and may even have protective properties or be less disruptive [12,13,14].

Reb A may be fermented by specific microbial populations, potentially supporting the growth of beneficial bacteria such as *Faecalibacterium*, a butyrate-producing, anti-inflammatory genus [3]. Reb A, alone or combined with prebiotics, increased *Akkermansia muciniphila* (a mucin-degrading bacterium) and *Bacteroides thetaiotaomicron* (butyrate-producing bacteria), contributing to a shift in the GM toward metabolically relevant profiles [3]. Others indicated that natural NNSs promoted the genus *Ruminococcus,* which plays a critical role in digesting complex carbohydrates and short-chain fatty acid (SCFA) production [15].

To date, most evidence regarding GM modulation has been derived from studies involving synthetic NNSs, while data on naturally derived, plant-based alternatives, such as reb A, have barely been evaluated. In addition, since the high-fat content affects the GM community, the aim of the present study was to examine the sucralose or reb A effects, at doses equivalent to the ADI, on the GM composition in rats fed a normal diet (ND) or a high-fat diet (HFD).

## 2. Materials and Methods

### 2.1. Study Design

This is a complementary study conducted within the framework of the original experiment. A detailed description of the research design is available in Ramos-García et al. (2021) [16]. Briefly, the experiment included two phases. In phase 1, animals were maintained for 8 weeks under the two diets explained above (ND and HCD). After this period, the animals under the HCD developed an altered glycemic response. In phase 2, the animals receive 8-week NNS interventions along with an ND or HFD. Before and after the NNS interventions, we performed an evaluation of the glycemic response of the animals. After, we selected the sucralose (SCL), reb A (REB), sucrose (SUC), and glucose (GLU) groups to investigate the effects on GM composition. See Supplementary Materials, Figure S1, for details.

### 2.2. Animals

Male Wistar rats weighing 150–200 g (6 to 8 weeks old) were provided by the Faculty of Medicine of the National Autonomous University of Mexico (UNAM). They were housed in polycarbonate cages (four per cage) with a 12 h light/dark cycle (7:00 h to 19:00 h), controlled temperature (18 °C to 26 °C), ventilation cycles (12 to 15 air changes per hour), and relative humidity (45% to 60%). All procedures were performed according to the Official Mexican Standard NOM-062-ZOO-1999 and the guidelines established by the Research Committee for the Care and Use of Laboratory Animals of the UNAM. The study protocol was approved by the Institutional Review Board of the Academic Division of Health Sciences, Juarez Autonomous University of Tabasco (002/CIP/DACS).

### 2.3. Diets

In phase 1, animals were randomly allocated into two groups to receive either an ND or a hypercaloric diet (HCD) for 8 weeks. The ND comprised standardized LabDiet Rodent^®^ 5001 chow (28.67% protein, 57.94% carbohydrate, 13.38% fat, 3.36 kcal/g) and pure water, whereas the HCD comprised the HFD (4.6 kcal/g of energy, 26.7% carbohydrates, 13.2% proteins, 60.0% fat) along with a 30% sucrose solution. The diets were administered in pellet form. Before the intervention phase, the rats underwent a one-week acclimation period, receiving the ND and pure water. All animals had ad libitum access to food and fluid over the experimental period.

### 2.4. Treatments

The following sweeteners were employed in this study: 1.3% sucralose (Sweeny^®^ plus, Salutare, S.A. de C.V., Estado de Mexico, Mexico), pure reb A (Anhui Minmetals, Hefei, China), glucose (Roquette corporation^®^, Lestrem, France), and sucrose (Zulka^®^, Sinaloa, Mexico). Each dietary group (ND and HCD) receive 4% glucose (GLU), 4% sucrose (SUC), sucralose (SCL) (5 mg/kg BW/d), or reb A (REB) (4 mg/kg BW/d expressed as steviol equivalents (*n* = 8 for each group) for 8 weeks. The sweeteners were administered in drinking water. The NNS concentrations were adjusted daily to provide the desired dose to each animal based on the average of daily fluid consumption per day and BW using an Excel fact sheet. To control the high glucose content in the commercial sweeteners, the 4% glucose solution was introduced as a primary control. The glucose concentrations were adjusted to 4% in the NNS groups. The SUC groups served as a secondary comparator. Food and fluid intake were recorded daily, and energy intake was calculated weekly. BW was measured twice a week using an electronic precision balance (Precision BJ 2200C). Throughout the NNS treatments, the animals did not change weekly body weight evolution, total weight gain, or energy intake under either the ND or HFD. Detailed information regarding the calculation and metabolic outcomes was previously reported [16]. After eight weeks, each animal was isolated for handling, and fresh fecal samples were collected in duplicate. In addition, plasma concentrations of glucose, insulin, and lipids were quantified. All samples were immediately stored at −80 °C for further analysis.

### 2.5. DNA Extraction and 16S rRNA PCR

The GM composition was assessed through the sequencing of the 16S rRNA gene in the fecal samples. Total DNA was isolated from the feces of the rats using the QIAamp^®^ Fast DNA Stool Mini Kit (QIAGEN, Hilden, Germany) (Cat No. 51604) following the manufacturer’s instructions. Purified DNA was quantified in duplicate using a Nanodrop 2000 (Thermo Fisher Scientific, Waltham, MA, USA). All the samples were stored at −80 °C until further analysis. The V3−V4 hypervariable regions of the bacterial 16S rRNA gene were targeted using the following specific universal bacterial primer pairs:

515F 5′–TCGTCGGCAGCGTCAGATGTGTATAAGAGACAGCCTACGGGNGGCWGCAG–3′.

806R 5′–GTCTCGTGGGCTCGGAGATGTGTATAAGAGACAGGACTACHVGGGTATCTAATCC–3′.

For each sample, 5 ng/µL of the purified DNA, 5 µL of forward primer (1 µM), 5 µL of reverse primer (1 µM), 12.5 µL of 2X Platinum SuperFi PCR Master Mix (Thermo Fisher Scientific, Waltham, MA, USA), and 10 µL of PCR-grade water, for a total volume of 25 µL, were used for the initial PCR reaction. The PCR reaction was performed on an Applied Biosystems GeneAmp 9700 96-well Thermal Cycler (Thermo Fisher Scientific, Waltham, MA, USA) using the following parameters: 98 °C for 3 min predenaturation, followed by 25 cycles of amplification consisting of denaturation (98 °C for 30 s), alignment (55 °C for 30 s), elongation (72 °C for 30 s), and a final elongation step (72 °C for 5 min). Amplicons were generated, cleaned, indexed, and sequenced according to the Illumina MiSeq 16S Metagenomic Sequencing Library Preparation Protocol [17].

### 2.6. Application of 16S rRNA Gene Sequencing

Libraries were demultiplexed using a dual-index approach with the Nextera XT Index kit (Illumina Inc., San Diego, CA, USA). Paired-end 2 × 250 bp reads were generated using the MiSeq Reagent Kit V2 (500 cycles). Truncated reads shorter than 75% of the original length (~150 bp) were discarded. Reads containing ambiguous characters (N bases) were removed. Only sequences with an overlap of at least 20 bp were assembled based on their overlapping regions. Reads that could not be successfully merged were discarded, which reduced the final sample size. Trimmomatic v. 0.36 software was used to process the raw fastQ files [18], and the mate-paired files were trimmed to dispose of bases prior to estimating the sequencing error. The aligned data were analyzed using quantitative insights into microbial ecology (QIIME) [19]. Dada2 and deblur denoising packages were used to match operational taxonomic units (OTUs) with 97% sequence similarity against the Silva database, release 138. All sequencing reactions were performed at the Sequencing Unit of the National Institute of Genomic Medicine in Mexico.

### 2.7. Data Analysis

Data were analyzed and plotted using Graphpad Prism Software version 7.0 (San Diego, CA, USA) or QIIME Software version 2.0 (San Diego, CA, USA). The Kruskal–Wallis test was employed to compare the differences in the α-diversity (Shannon index) across different treatments. Permutational multivariate analysis of variance (PERMANOVA), with Bray–Curtis dissimilarity and principal component analysis (PCoA), was used to analyze β-diversity. The ratio of Firmicutes/Bacteroidetes (F/B ratio) was calculated by dividing the relative abundance of *Firmicutes* by that of *Bacteroidetes*. The differences in the relative abundances of bacterial taxa and the F/B ratio between sweeteners were compared using one-way analysis of variance (ANOVA) for multiple comparisons or the Kruskal–Wallis nonparametric equivalent test with Tukey’s or Dunn’s post hoc test, respectively. A two-tailed Student’s t or Mann–Whitney test was used to analyze the differences between the diets. Data were expressed as media ± standard error of the mean (SEM), unless otherwise specified. Differences were considered statistically significant at *p* < 0.05.

## 3. Results

### 3.1. Effect of Sucralose or Reb A on GM Diversity

**Figure 1:**
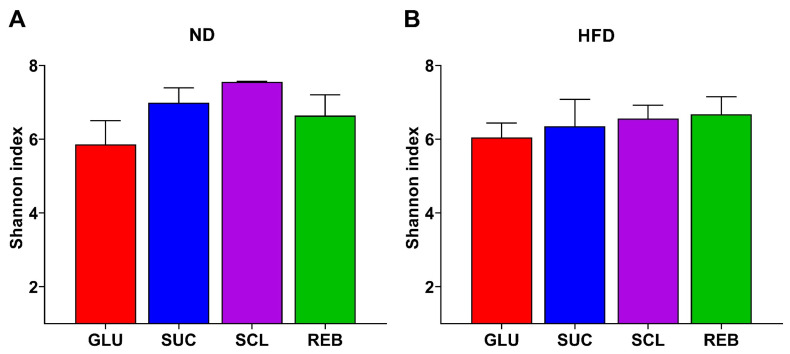
Effect of NNSs on the α-diversity (Shannon Index) of the GM in (**A**) ND- or (**B**) HFD-fed rats. Data are presented as mean ± SEM. Statistical analyses were performed using one-way ANOVA and the post hoc Tukey’s test. ND—normal diet, HFD—high-fat diet, GLU—glucose, SUC—sucrose, SCL—sucralose, and REB—reb A.

Figure 1 shows the α-diversity index of the GM of the rats consuming SCL and REB compared to the controls (GLU and SUC) under the ND and HFD. No significant differences were found in the α-diversity between the NNS treatments in the ND or HFD groups (*p* > 0.05) (Figure 1, panels A and B). Statistical data for α-diversity based on the Kruskal–Wallis test are shown in Supplementary Materials, Table S1.

**Figure 2:**
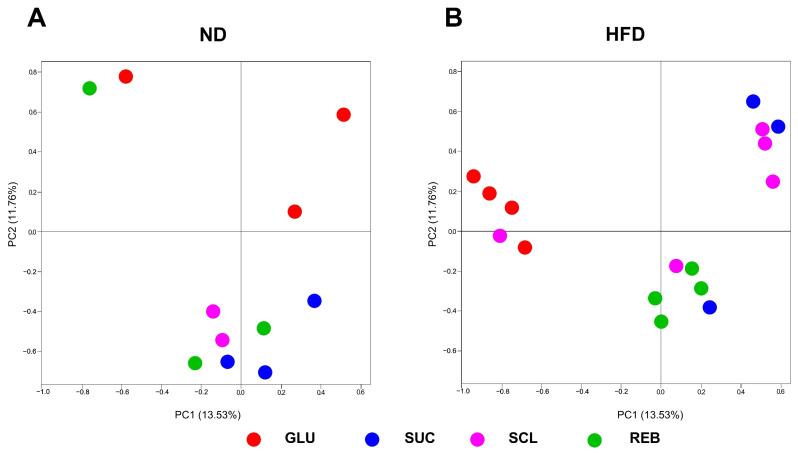
Effects of NNSs on the β-diversity (Bray–Curtis index) of the GM in rats fed with (**A**) the ND, or (**B**) the HFD. PCoA of the bacterial 16S rRNA examining phylogenetic distances between treatments. No differences were found in the PERMANOVA analysis. ND—normal diet, HFD—high-fat diet, GLU—glucose, SUC—sucrose, SCL—sucralose, and REB—reb A.

PCoA distances indicated that neither SCL nor REB altered the microbe structure in the ND- and HFD-fed rats. SUC consumption with the ND and SCL consumption with the HFD tended to cluster distinctly from the GLU treatment; however, the *p*-adjust does not support these differences (PERMANOVA, *p* = 0.02 and *p*-adjust = 0.16). The variation in the dataset was explained by two principal components, with a cumulative variance of 25.29%. Figure 2, panels A, B, and C, shows the comparisons between the NNSs and between diets. Supplementary Materials, Table S2 displays the statistical data of the PERMANOVA.

### 3.2. Effect of Sucralose or Reb A on GM Composition

#### 3.2.1. Phylum Level

**Figure 3:**
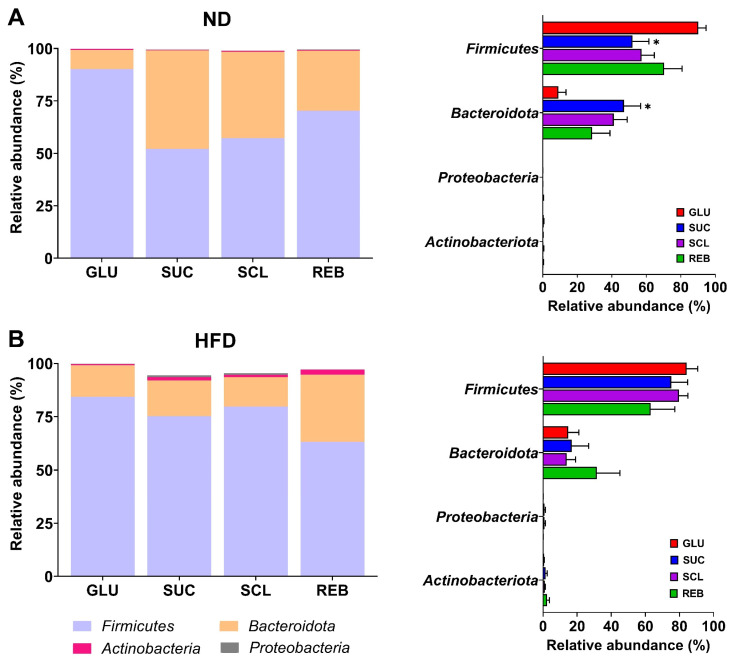
Distribution of the four dominant phyla in the GM between treatments in the (**A**) ND and (**B**) HFD groups. Data are expressed as relative abundance (%). Statistical analyses were performed using one-way ANOVA and the post hoc Tukey’s test. ND—normal diet, HFD—high-fat diet, GLU—glucose, SUC—sucrose, SCL—sucralose, and REB—reb A. * *p* < 0.05 vs. GLU.

Figure 3 shows the distribution of the four dominant phyla between the treatments in the ND and HFD groups. The dominant phylum across all treatments in both the ND- and HFD-fed rats were *Firmicutes* (ND: 67.44 ± 5.06%; HFD: 75.59 ± 4.72%) and *Bacteroidetes* (ND: 31.49 ± 5.07%; HFD: 19.26 ± 4.60%), with a lower proportion of *Actinobacteria* (ND: 0.38 ± 0.06%; HFD: 1.38 ± 0.40%) and *Proteobacteria* (ND: 0.105 ± 0.04%; HFD: 0.47 ± 0.21%).

In the ND-fed rats, SUC (52.08 ± 9.40%, *p* = 0.02) decreased *Firmicutes* and increased *Bacteroidota* (47.05 ± 9.59%, *p* = 0.02) relative to GLU (90.16 ± 4.40% and 9.09 ± 4.34%, respectively) (Figure 3A). In the HFD-fed rats, the presence of *Actinobacteria* and *Proteobacteria* was observed in smaller proportions, but without reaching statistical significance between treatments (Figure 3B). Supplementary Materials, Table S3 displays the statistical data of the GM composition at the phylum level following the NNS treatments.

#### 3.2.2. F/B Ratio

**Figure 4:**
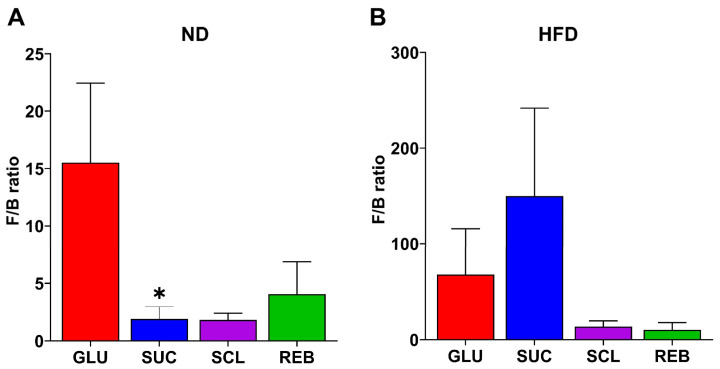
Effect of sucralose and reb A on the F/B ratio of the GM in rats fed with the (**A**) ND or (**B**) HFD. Data are expressed as relative abundance (%) and presented as media ± SEM. Statistical analysis between sweeteners was performed using one-way ANOVA or Kruskal–Wallis’s test with the post hoc Tukey’s or Dunn’s test, respectively. * *p* < 0.05 vs. GLU. ND—normal diet, HFD—high-fat diet, GLU—glucose, SUC—sucrose, SCL—sucralose, and REB—reb A.

The F/B ratio has been reported to influence the maintenance of gut homeostasis and the onset of several pathologies [20]. Our analysis showed that in the ND-fed rats, only the SUC group had a lowered F/B ratio [0.88 (0.54, 3.77)] compared to the GLU group [17.52 (5.17, 94.87)] (*p* = 0.038) (Figure 4A). However, neither in the HFD-fed rats nor in the ND-fed rats were significant differences observed between the NNS treatments (Figure 4B).

#### 3.2.3. Class Level

**Figure 5:**
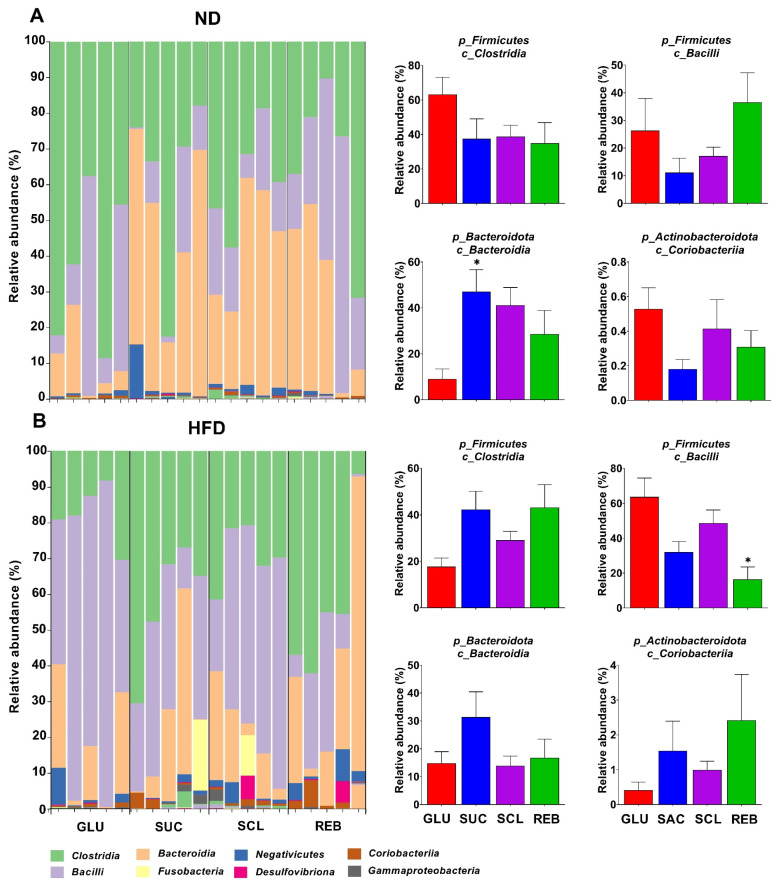
Effect of NNSs on the GM composition, at the class level, in rats fed with (**A**) the ND or (**B**) the HFD. Data are expressed as relative abundance (%) and presented as mean ± SEM. Statistical analyses were performed using one-way ANOVA with the post hoc Tukey’s test. * *p* < 0.05 vs. GLU. ND—normal diet, HFD—high-fat diet, GLU—glucose, SUC—sucrose, SCL—sucralose, and REB—reb A.

Figure 5 displays the distribution of the GM at the class level between the NNS treatments in the ND and HFD groups. We observed that in the ND-fed rats, SUC consumption (47.05 ± 9.59%) induced an increase in the abundance of *Bacteroidia* compared to GLU consumption (9.09 ± 4.34%, *p* = 0.024) (Figure 5A). In the HFD-fed rats, REB (16.45 ± 7.11%) decreased the media proportions of *Bacilli* in comparison with GLU (63.72 ± 10.74%, *p* = 0.003) (Figure 5B). No significant changes were identified with the consumption of NNSs in the other bacterial classes (*p* > 0.05).

#### 3.2.4. Genera Level

**Figure 6:**
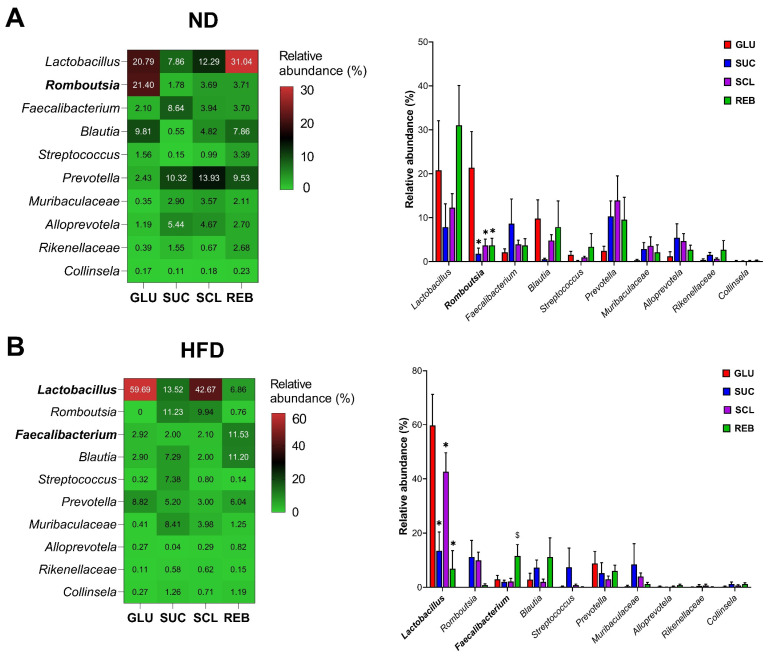
Distribution of the main genera of the GM after NNS consumption in the rats fed with (**A**) the ND or (**B**) the HFD. Data are expressed as relative abundance (%). Statistical analyses were performed using one-way ANOVA with the post hoc Tukey’s test. * *p* < 0.05 vs. GLU and $ *p* < 0.01 vs. SUC. ND—normal diet, HFD—high-fat diet, GLU—glucose, SUC—sucrose, SCL—sucralose, REB—reb A.

The major genera were found in the *Firmicutes*, *Bacteroidetes*, and *Actinobacteria* phyla. In the rats fed with the ND, SCL (3.60 ± 1.41%), REB (3.71 ± 1.61%), and SUC (1.78 ± 1.29%) significantly reduced the relative abundance of the genus *Romboutsia* (phylum: *Firmicutes*; family: *Peptostreptococcaceae*) compared to GLU (21.40 ± 8.23%, *p* < 0.05) (Figure 6A). In the HFD rats, SCL (42.67 ± 6.98%) and REB (6.85 ± 6.67%) reduced *Lactobacillus* (phylum: *Firmicutes*; family: *Lactobacillaceae*) with respect to GLU (59.69 ± 11.51%). We noted that REB (11.53 ± 4.22%) increased *Faecalibacterium* (phylum: *Firmicutes*; family: *Ruminococcaceae*) compared to SUC (1.99 ± 0.68%, *p* < 0.01) (Figure 6B). In the other genera, we did not observe statistical significance.

### 3.3. Effect of the HFD on Diversity, Composition, and the F/B Ratio

**Figure 7:**
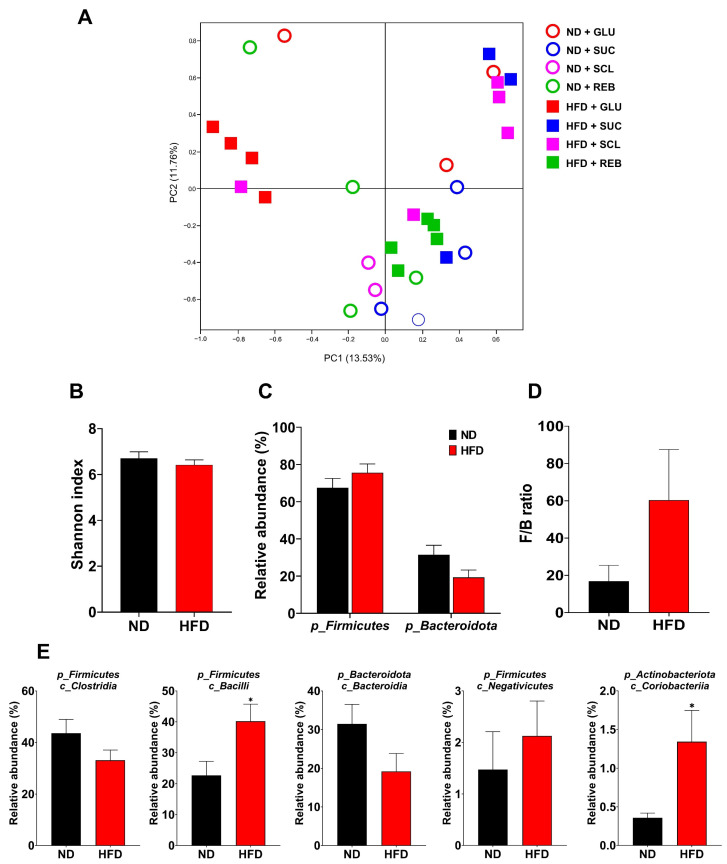
Effect of the HFD on diversity (**A**,**B**), composition at the phylum level (**C**), the F/B ratio (**D**), and the most abundant classes (**E**) of the GM. Data are expressed as relative abundance (%) and presented as mean ± SEM or median (p25, p70). Statistical analyses were performed using the Unpaired Student *t* test or the Mann–Whitney test. * *p* < 0.05. ND—normal diet, HFD—high-fat diet, GLU—glucose, SUC—sucrose, SCL—sucralose, and REB—reb A.

The effect of the HFD on the GM was evaluated regardless of the type of sweetener. Figure 7 shows the effects of the HFD on GM diversity, composition, and F/B ratio. With the consumption of the HFD, no significant effects were identified in the β-diversity (Figure 7A), α-diversity (Shannon index, Figure 7B), composition at the phylum level (Figure 7C), or F/B index (*p* > 0.05) (Figure 7D). At the class level, the HFD increased the proportions of *Bacilli* [40.30% (21.24, 74.54)] (*p* = 0.03) and *Coriobacteriia* [0.62% (0.24, 1.80)] (*p* = 0.02)] relative to the ND [16.68% (8.14, 28.29) and 0.29% (0.15, 0.50), respectively] (Figure 7E). A potential reduction in *Clostridia* was observed; however, no statistical significance was reached. At the genus level, the HFD did not induce significant modifications on *Lactobacillus*, *Romboutsia*, or *Faecalibacterium* (*p* > 0.05).

### 3.4. Effect of the HFD on Fasting Biochemical Parameters

In our previous publication, we found that neither in ND- nor HFD-fed rats did SCL, REB, or other NNSs produce significant effects on fasting glucose, total cholesterol, HDL-cholesterol, insulin levels, or HOMA-IR when compared to the glucose control [16]. In the present complementary study, we conducted a reanalysis to evaluate the impact of the HFD on fasting biochemical parameters (Table 1). When comparing the two diets, the HFD did not significantly alter the fasting glucose concentration with any of the NNSs, despite the evident glucose intolerance observed in the HFD group. Although lower fasting glucose and insulin levels were observed in the HFD-fed rats treated with SCL or REB, these differences did not reach statistical significance when compared to their ND-fed counterparts.

However, we observed some significant effects on the fasting lipid concentrations. HFD + SCL decreased total cholesterol (53.0 ± 3.56 mg/dL, *p* = 0.0450) and HDL-cholesterol (19.65 ± 1.04 mg/dL, *p* = 0.0073) in comparison to the ND (64.5 ± 3.81 and 24.89 ± 1.30 mg/dL, respectively), whereas HFD + REB decreased HDL-cholesterol (20.47 ± 1.03 mg/dL, *p* = 0.0013) compared with the ND (25.61 ± 0.73 mg/dL). Additionally, as expected, HFD + SUC increased total cholesterol (65.63 ± 4.57 mg/dL, *p* = 0.0143) and triglycerides (62.63 ± 5.81 mg/dL, *p* = 0.0101) with respect to the ND (49.63 ± 3.44 and 41.25 ± 4.22 mg/dL, respectively).

## 4. Discussion

In the present study, neither the relative abundance of the dominant phyla in the GM nor the F/B ratio was altered after 8 weeks of consuming the recommended doses of sucralose or reb A, which suggests that these NNSs did not cause substantial changes in the GM composition. In addition, no changes were observed in the GM α- and β-diversity. Collectively, our results agree with previous studies that evaluated sucralose or reb A in animal models that ingested similar diets [21]. Many of the acute studies on the biological fate of sucralose have shown that this substance is not absorbed, but it is eliminated unchanged in feces, which makes it unlikely to be a substrate for the GM [22,23]. Reb A is known to resist hydrolysis by pancreatic or brush border enzymes, but it is converted into aglycone steviol and glucose by gut microbes expressing β-glucosidases in the gastrointestinal tract [1,24]. After degradation, steviol is absorbed and transformed into steviol glucuronide [21], while glucose is mainly absorbed by enterocytes and has limited effects on glucose homeostasis [25].

Alterations in the F/B ratio have frequently been examined in the context of obesity; however, its reliability as a biomarker remains highly debated. Although early studies suggested that an increased F/B ratio might be associated with obesity [26], subsequent research reported a decreased ratio or no effects between obese and lean individuals or animal models [27,28,29,30]. Moreover, focusing solely on the F/B ratio may oversimplify the complex and dynamic nature of the microbial community structure and its metabolic interactions with the host. Therefore, while the F/B ratio can provide a general overview of community shifts, it should be interpreted with caution and in conjunction with more specific taxa-level or functional analyses to better understand microbiota contributions to obesity and metabolic health. Here, we did not find effects of NNSs on this outcome.

At the class level, exposure to reb A decreased the abundance of *Bacilli* under the HFD, which could be considered beneficial since the increase in this genus has been associated with alterations in metabolism [31]. Sucralose and reb A decrease the genus *Romboutsia* in the rats fed the ND. These bacteria can utilize carbohydrates, ferment various amino acids, and modulate lipid metabolism, suggesting that their reduction could negatively influence host metabolic homeostasis [32]. Additionally, both sweeteners significantly reduced the *Lactobacillus* abundance in the HFD-fed rats. This genus plays a key role in maintaining intestinal barrier integrity, modulating immune responses, and producing SCFAs that regulate inflammation and lipid metabolism [33]. The decline in *Lactobacillus* observed here aligns with prior studies reporting stevia glycosides’ inhibitory effects on *Lactobacilli* growth, particularly reb A [24,33,34]. This microbial alteration may contribute to the observed dysregulation of lipid metabolism in the HFD-NNS groups, potentially exacerbating low-grade inflammation and impairing metabolic and immune functions. These shifts in the GM highlight possible adverse implications for host metabolic health and immune homeostasis. In our reanalysis of fasting biochemical parameters, we found that SCL induced a decrease in total cholesterol and HDL-cholesterol, while REB decreased HDL-cholesterol. This could be associated with lower synthesis of SCFAs, bile salt deconjugation, and metabolic receptor modulation [35,36]. Further studies should be carried out to evaluate the influence of these specific NNSs on widely utilized probiotic strains in humans and for a more prolonged time.

When comparing the two types of diets, the HFD regimen showed modifications in the GM at the class level, increasing *Bacilli*, but no significant effects on the F/B ratio were observed. The absence of significant effects of the HFD and NNSs on the F/B ratio could be mainly explained by the simultaneous reduction in *Clostridia*. It has been proposed that certain bacterial groups, such as *Bacilli*, can metabolize lipids, which could represent the main mechanism for the observed changes in our findings [37,38].

Our microbiota results are in line with the absence of effects of NNSs on the glycemic response, body weight, or energy intake in healthy rats and with the altered glycemic response reported in our previous research [16]. In that study, animals on the HCD developed altered glucose tolerance during phase 1, even though fasting glucose concentrations remained unchanged. We found that following the interventions with SCL or REB, no additional glycemic alterations were observed. Consistently, in this study, the GM analysis supported these findings, revealing no additional changes associated with these metabolic alterations. Contrariwise, other studies on male rodents have shown GM alterations. Acesulfame K and saccharin, administered via gavage (4 weeks) or beverages (11 weeks) at 2.5 or 650 times the ADI, respectively, induced frank GM changes, including the over-representation of *Bacteroides*, *Anaerostipes*, and *Sutterella*, or the under-representation of *Clostridiales* [8,39]. Notably, these interventions utilized doses surpassing admissible limits for humans, and although significant changes were observed, the used doses may not be reflective of real daily consumption.

In a recent investigation, a low dose (0.0003 mg/mL equivalent to 0.001 ADI) of sucralose over 16 weeks led to an increase in *Tenacibaculum*, *Ruegeria*, *Staphylococcus*, and *Allobaculum* at the genus level in the jejunum, ileum, and colon of mice [9]. Similarly, alterations in bacterial genera were observed in C57BL/6 mice after 6 months of sucralose at the human ADI, including an increase in *Ruminococcaceae* and *Ruminococcus* and a reduction in *Dehalobacterium*, *Lachnospiraceae*, *Anaerostipes*, and *Lachnospiraceae Ruminococcus* [2]. These differences, in comparison with the present study, may be partially explained by species differences and the length of the intervention.

Our study presents several strengths: a rat model of altered glycemic response induced by an HFD; the inclusion of both natural and artificial NNSs; the use of doses equivalent to the ADI; the incorporation of glucose and sucrose as control groups; and comparisons of NNS effects under two distinct dietary conditions. However, some limitations include the small sample size and the inability to evaluate the mechanisms by which dietary patterns affect the GM. Additionally, although the use of 16S rRNA gene sequencing constitutes a reliable method to study the diversity and composition of the microbiota, the relative bacterial abundances may not be reflect absolute changes; therefore, we suggest that future investigations include multiomics approaches to better understand the mechanisms, metabolites, and gene or protein expression involved in the interaction between these NNSs and the GM.

## 5. Conclusions

Collectively, our study indicates that when sucralose or reb A is consumed at recommended doses, there is no observed alteration in the diversity and composition of the GM at the phylum level. Consequently, our data suggest that these NNSs do not have a substantial impact on the GM. We recommend further research to delve into the potential modifications of the GM at the level of specific bacterial taxa under different types of diets, as this could offer valuable insights for clinical interventions. In addition, extending this research to involve human participants and including a broader range of microbial analyses will be essential steps in translating these findings into clinical practice.

## Figures and Tables

**Table 1:** Effect of the HFD on fasting biochemical parameters.

Parameter	GLU		SUC		SCL		REB	
	ND	HFD	ND	HFD	ND	HFD	ND	HFD
Glucose (mg/dL)	123.3 ± 6.9	111.4 ± 9.8	127.9 ± 9.9	125.6 ± 6.7	120.4 ± 12.3	126.3 ± 14.9	137.9 ± 12.4	123.3 ± 8.5
Insulin (µU/mL)	79.3 ± 24.3	56.5 ± 12.9	72.5 ± 20.4	86.4 ± 25.4	102.3 ± 27.8	65.6 ± 36.1	76.3 ± 29.9	28.1 ± 19.2
HOMA-IR (mg/dL × µU/mL)	26.5 ± 9.0	16.9 ± 4.7	25.6 ± 8.2	28.8 ± 9.9	30.8 ± 8.00	29.3 ± 20.5	29.5 ± 13.4	8.44 ± 5.8
Total Cholesterol (mg/dL)	51.4 ± 4.9	51.0 ± 3.7	49.6 ± 3.4	65.6 ± 4.6 *	64.5 ± 3.8	53.0 ± 3.6 *	65 ± 2.9	55.5 ± 4.8
Triglycerides (mg/dL)	38.6 ± 3.0	48.1 ± 3.5	41.2 ± 4.2	62.6 ± 5.8 *	45 ± 1.7	52.6 ± 5.5	48.2 ± 4.2	52.8 ± 3.0
HDL-Cholesterol (mg/dL)	21.0 ± 1.4	18.5 ± 0.8	20.9 ± 0.7	24.3 ± 2.1	24.9 ± 1.3	19.6 ± 1.0 *	25.6 ± 0.7	20.5 ± 1.0 *

Values are mean ± SEM. HFD, high-fat diet; ND, normal diet; GLU, glucose; SUC, sucrose; SCL, sucralose; REB, reb A. HOMA-IR, homeostatic model assessment. The Unpaired Student *t* test or Mann–Whitney test was used. Asterisks indicate significant differences in the comparisons between diets (i.e., HFD vs. ND with a *p* < 0.05) per sweetener.

## Data Availability

The raw data supporting the conclusions of this article will be made available by the authors on request.

## Supplementary Materials

The following supporting information can be downloaded at: https://www.mdpi.com/article/10.3390/metabo15080529/s1, Figure S1: Experimental design for this GM secondary analysis adapted from Ramos-García et al. (2021) [16]; Table S1: Diversity analysis following treatments with NNSs compared to GLU or SUC under the two different dietary conditions; Table S2: PERMANOVA on the Bray-Curtis distance for the structure of the fecal microbiota between treatments (permutations = 9999) in rats with ND or HFD; Table S3. Composition of the gut microbiota at phylum level following NNS treatments compared to GLU or SUC under the two different dietary conditions.

## Abbreviations

The following abbreviations are used in this manuscript:

NNS	Non-nutritive sweeteners
ADI	Acceptable daily intake
FDA	Food and Drug Administration
GM	Gut microbiota
SCL	Sucralose
REB	Rebaudioside A
GLU	Glucose
SUC	Sucrose
ND	Normal diet
HFD	High-fat diet
HCD	Hypercaloric diet
F/B ratio	Firmicutes/Bacteroidetes ratio
BW	Body weight
