# Supplementary material for: Gut Microbiota Composition in Rats Consuming Sucralose or Rebaudioside A at Recommended Doses Under Two Dietary Interventions
Source: Metabolites. 2025 Aug 4;15(8):529. doi: 10.3390/metabo15080529 (PMC12388177; doi:10.3390/metabo15080529)
Supplement: Supplementary file 1 [file metabolites-15-00529-s001.zip › metabolites-3740327-supplementary.pdf]

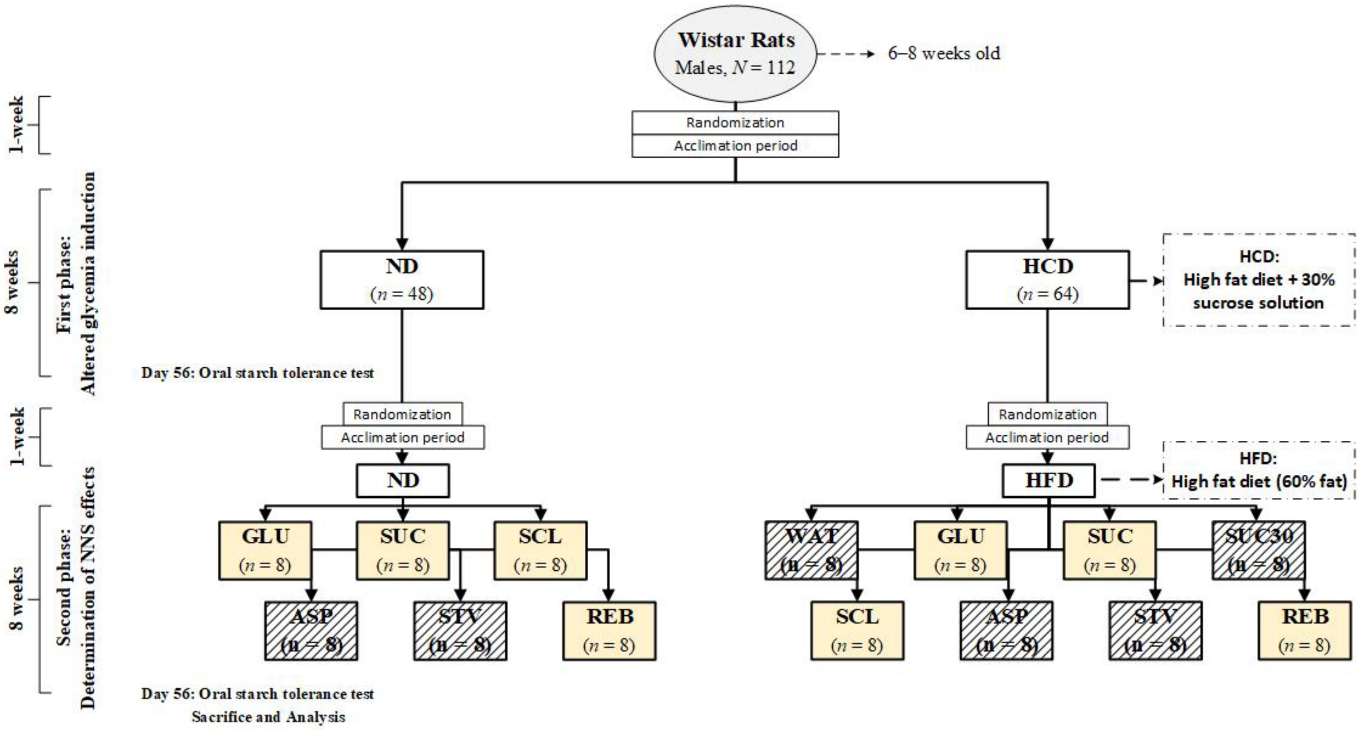

Figure S1. Experimental design for this GM secondary analysis adapted from Ramos-García et al. (2021). Yellow boxes denote all the selected groups for this study, including NNSs (SCL, REB) and control groups (GLU, SUC). Excluded groups are depicted using boxes with a diagonal hatching pattern.

Table S1. Diversity analysis following treatments with NNSs compared to GLU or SUC under the two different dietary conditions.

**Comparison**	**ND**			**HFD**		
	n	***p***	***q***	***n***	***p***	***q***
GLU *vs* SUC	4	0.15	0.70	3	0.48	0.74
GLU *vs* SCL	2	0.08	0.70	5	0.22	0.71
GLU *vs* REB	4	0.47	0.74	4	0.24	0.71

Data were analyzed using the Kruskal-Wallis test to compare the differences in α-diversity (Shannon index) between the different treatments in rats fed with ND or HFD. No statistically significant differences were found. *ND* normal diet; *HFD* high-fat diet; *GLU* glucose; *SUC* sucrose; *SCL* sucralose; *REB* reb A

Table S2. PERMANOVA on the Bray-Curtis distance for the structure of the fecal microbiota between treatments (permutations = 9999) in rats with ND or HFD.

**Comparison**	**ND**				**HFD**			
	n	**Pseudo-*F***	***p***	***q***	***n***	**Pseudo-*F***	***p***	***q***
GLU vs. SUC	7	2.74	0.02	0.16	7	1.37	0.18	0.27
GLU vs. SCL	5	1.81	0.19	0.27	9	1.90	0.02	0.16
GLU vs. REB	7	1.18	0.24	0.29	8	1.57	0.06	0.17

*p <0.05 vs. GLU. *ND* normal diet; *HFD* high-fat diet, *GLU* glucose, *SUC* sucrose, *SCL* sucralose, *REB* reb A, Pseudo-*F*, *F* value by permutation

Table S3. Composition of the gut microbiota at phylum level following NNSs treatments compared to GLU or SUC under the two different dietary conditions.

Phylum	ND				HFD			
	GLU	SUC	SCL	REB	GLU	SUC	SCL	REB
Firmicutes	90.16 ± 4.40	52.08 ± 9.40*	57.26 ± 7.354*	70.28 ± 10.47	84.29 ± 6.50	75.21 ± 9.64	79.69 ± 5.29	63.15 ± 14.09
Bacteroidetes	9.097 ± 4.34	47.05 ± 9.59*	41.15 ± 7.74*	28.65 ± 10.28	14.82 ± 6.23	16.82 ± 9.95	13.90 ± 5.22	31.50 ± 13.64
Actinobacteria	0.54 ± 0.12	0.23 ± 0.08	0.436 ± 0.15	0.32 ± 0.09	0.56 ± 0.24	1.542 ± 0.85	1.025 ± 0.26	2.43 ± 1.31
Proteobacteria	0.00 ± 0.00	0.06 ± 0.04	0.07 ± 0.03	0.28 ± 0.14	0.11 ± 0.03	0.90 ± 0.55	0.82 ± 0.63	0.05 ± 0.05

Groups with significant differences are in bold for easier identification

Data are expressed as mean ± SEM. The *p*-values were obtained from comparisons based on one-way multiple comparisons ANOVA corrected by false discovery rate (FDR). *$p < 0.05$ vs GLU. Data are expressed as percentage. *ND* normal diet; *HFD* high-fat diet; *GLU* glucose; *SUC* sucrose; *SCL* sucralose; *REB* reb A
